# Medication Reviews and Clinical Outcomes in Persons with Dementia: A Scoping Review

**DOI:** 10.3390/pharmacy11050168

**Published:** 2023-10-20

**Authors:** Rishabh Sharma, Neil Mahajan, Sarah Abu Fadaleh, Hawa Patel, Jessica Ivo, Sadaf Faisal, Feng Chang, Linda Lee, Tejal Patel

**Affiliations:** 1 School of Pharmacy, University of Waterloo, 10 Victoria St S A, Kitchener, ON N2G 1C5, Canada; r367shar@uwaterloo.ca (R.S.); sfadaleh@gmail.com (S.A.F.); h267patel@uwaterloo.ca (H.P.); jarivo@uwaterloo.ca (J.I.); sadaf.faisal@uwaterloo.ca (S.F.); feng.chang@uwaterloo.ca (F.C.); 2 Faculty of Health, Western University, 1151 Richmond St, London, ON N6A 5B9, Canada; neil.mahajan1234@gmail.com; 3 CFFM MINT Memory Clinic, 25 Joseph St, Kitchener, ON N2G 4X6, Canada; lee.linda.lw@gmail.com; 4 Schlegel-UW Research Institute for Aging, 250 Laurelwood Dr, Waterloo, ON N2J 0E2, Canada; 5 Department of Family Medicine, McMaster University, 100 Main St W 5th Floor, Hamilton, ON L8P 1H6, Canada

**Keywords:** older adults, dementia, medication review, drug-related problems

## Abstract

Persons diagnosed with dementia are often faced with challenges related to polypharmacy and inappropriate medication use and could benefit from regular medication reviews. However, the benefit of such reviews has not been examined in this population. Therefore, the current scoping review was designed to identify the gaps in the current knowledge regarding the impact of medication reviews on the clinical outcomes in older adults with dementia. Relevant studies were identified by searching three databases (Ovid MEDLINE, Ovid EMBASE, and Scopus) from inception to January 2022 with a combination of keywords and medical subject headings. After the removal of duplicates and ineligible articles, 22 publications of the initial 8346 were included in this review. A total of 57 outcomes were identified, including those pertaining to the evaluation of medication use (n = 17), drug-related interventions (n = 11), drug-related problems (n = 10), dementia-related behavioral symptoms (n = 8), cost-effectiveness (n = 2), drug-related hospital admissions (n = 1), as well as outcomes classified as other (n = 7). Gaps identified through this scoping review included the paucity of studies measuring the impact of medication reviews on the medication management capacity and medication adherence, quality of life, and mortality.

## 1. Introduction

Dementia is an umbrella terms that encapsulated a number of neurodegenerative, irreversibly progressive disorders that are marked by cognitive decline and a steady reduction in everyday function, and it is typically accompanied by behavioral issues [1]. Cognitive impairment (CI) or dementia affects the ability to learn, memory, reasoning, focus, understanding, language, and judgment. Given that the risk of being diagnosed with dementia increases with age, the global prevalence of dementia is expected to increase from 50 to 150 million by 2050, with the aging of the world population [2,3,4]. Dementia is presently the seventh leading cause of death, and it is one of the primary causes of impairment and dependency in older people worldwide [2]. People with dementia and their caregivers, family, and society at large all experience social, psychological, physical, and financial repercussions. In Canada, the annual healthcare cost of dementia, including the out-of-pocket cost of caring for people with dementia, was CAD 10.4 billion in 2016 [5,6]. Older adults who have dementia commonly experience coexisting medical conditions, including hypertension, diabetes mellitus, coronary artery disease, stroke, and heart failure. These comorbidities are highly prevalent among this population [7]. Older adults who have CI or dementia are particularly at risk for drug-related problems (DRPs), with 41% of hospital admissions in older adults with dementia thought to be partially or entirely related to DRPs, which is higher than older adults without dementia [8]. Older adults with dementia have more comorbid conditions and are often prescribed multiple medications, which further increases the risk of DRPs [9]. Studies have reported that more than half of older adults with dementia are prescribed five or more medications per day [8]. The use of multiple medications, or polypharmacy, in older adults with dementia was found to be associated with the use of potentially inappropriate medications (PIMs), which are medications that increase the risk of adverse events. The literature reports the higher prevalence of PIMs among older adults with dementia, ranging between 10.2 and 63.4% [10,11,12,13]. Additionally, managing medications in people with dementia may lead to drug-related hospital admissions, medication mistakes, and dependency on others to help with medication management responsibilities [14]. Adherence to a prescribed regimen can be very difficult for older adults with dementia due to complex medication regimens, memory loss, and other cognitive deficits [15]. Polypharmacy, complex medication regimens, and the use of PIMs in older adults with dementia are associated with an increased risk of adverse events and drug interactions, medication nonadherence, an increased risk of hospitalization or prolonged hospitalization, and economic burden on patients and the healthcare system [16,17]. Moreover, prescribing decisions made for older adults with dementia lack unbiased scientific evidence, as this population has been excluded from 85% of the clinical trials [18].

Optimizing medications in elderly individuals with dementia is a crucial step in addressing the complexity of prescribing medication and changing the treatment goals as the illness advances [19,20]. Regular reviews of medications could potentially address this concern. Pharmaceutical Care Network Europe (PCNE) states that a medication review is a structured assessment of a patient’s medications to optimize medicine usage and enhance health outcomes [21]. Medication reviews include several components, such as an assessment of the medications prescribed regularly and a review of medical information such as laboratory workups, diagnostic imaging from the medical records, and an interview with the patient to identify DRPs and implement interventions to address them [22]. Several clinical trials and observational studies have been conducted to evaluate the effectiveness of medication reviews in persons with dementia. However, there is a high degree of variability in the methodologies and outcomes examined. Therefore, the aim of our scoping review is to identify gaps related to the impact of medication reviews conducted in older adults with dementia on DRPs and clinical outcomes.

## 2. Materials and Methods

The foundation for the conduct of this scoping review was the 5-stage framework developed by Arksey and O’Malley. We also used the PRISMA Extension for Scoping Reviews (PRISMA—ScR) to report the results [23,24]. We followed the five steps recommended by Arksey and O’Malley to conduct the scoping review, first, by identifying the research question; second and third, by identifying and selecting the relevant studies for inclusion in the review; fourth, by charting the data; and fifth, by collating, summarizing, and reporting the results.

### 2.1. Step 1: Identifying the Research Question

As stated before, this scoping review was conducted to identify gaps in the current knowledge regarding the impact of medication reviews on clinical outcomes, and to identify the different types of DRPs reported in older adults with dementia. Pharmacists could conduct medication reviews of people with dementia on their own or with a multidisciplinary team of people.

### 2.2. Step 2: Identifying the Relevant Studies

A single reviewer (R.S.) prepared a comprehensive search strategy with the help of a research librarian. Ovid EMBASE, Ovid MEDLINE, and Scopus were searched from inception to January 2022. The search terms used in each database included a combination of medical subject headings and keywords (limited to title, abstract, and keywords) related to medication reviews, older adults, and dementia and linked by the Boolean operators (AND, OR), as shown in Table S1. Advanced search options, such as truncation use on keywords where appropriate, subject heading explosion, and adjacency features, were used based on the database functionality. Results were exported from each database into Microsoft^®^ Excel^®^ (Office 365 ProPlus Version 1906), where duplicates were removed.

### 2.3. Step 3: Study Selection

The first 520 articles were screened by two reviewers (R.S. and H.P.) to establish the inter-rater reliability in screening between the two researchers. Given the strength of the inter-rater reliability (Kappa coefficient of 0.92), the two reviewers independently screened 50% of the remaining article titles and abstracts. The bibliographies of the pertinent studies were also screened for additional relevant studies. The studies were included if (1) participants were older adults (age ≥ 55 years) diagnosed with dementia and/or cognitive impairment; (2) they were referenced as medication reviews. Studies were excluded if (1) patient participants were not older adults (aged <55 years); (2) patient participants were older adults but not diagnosed with dementia; (3) they included non-human populations; (4) they were published in a non-English language; and (5) they were editorials, commentaries, opinions, letters to the editor, systematic reviews or meta-analyses, or case reports.

### 2.4. Step 4: Data Charting

Data extraction from the included studies was carried out using a Microsoft^®^ Excel^®^ spreadsheet, specifically the Office 365 ProPlus Version 1906. The following data were abstracted: the study design (qualitative/quantitative studies/randomized controlled trials (RCTs), non-RCTs, retrospective studies) and study details (study population demographics, year of publication, country, publication year, intervention details, sample size, DRPs identified, recommendations accepted to resolve DRPs, inclusion/exclusion criteria, study outcomes, and results). Data abstractions were completed by two reviewers (R.S. and N.M.) independently, after which they were compared to ensure accuracy, consistency, and completeness.

### 2.5. Step 5: Collating, Summarizing, and Reporting the Results

The following data were collected and summarized: demographic data; characteristics of the studies, including the study design, year of publication, and country of origin; and the effectiveness of the medication review. Additionally, the review encompassed an evaluation of the medication effectiveness, incorporating both quantitative data and narrative descriptions. This comprehensive approach allowed for a thorough assessment of the research findings. Results were categorized and summarized based on the clinical outcomes reported in terms of identifying DRPs, types of DRPs, changes in the number of prescribed medications, recommendations to resolve DRPs, and reductions in drug usage, mortality, and hospital admissions among older adults with dementia [25,26,27,28].

The types of care settings [29], pharmacist care interventions [30], DRPs, and drug-related interventions (DRIs) are defined in Table S2.

## 3. Results

**Figure 1:**
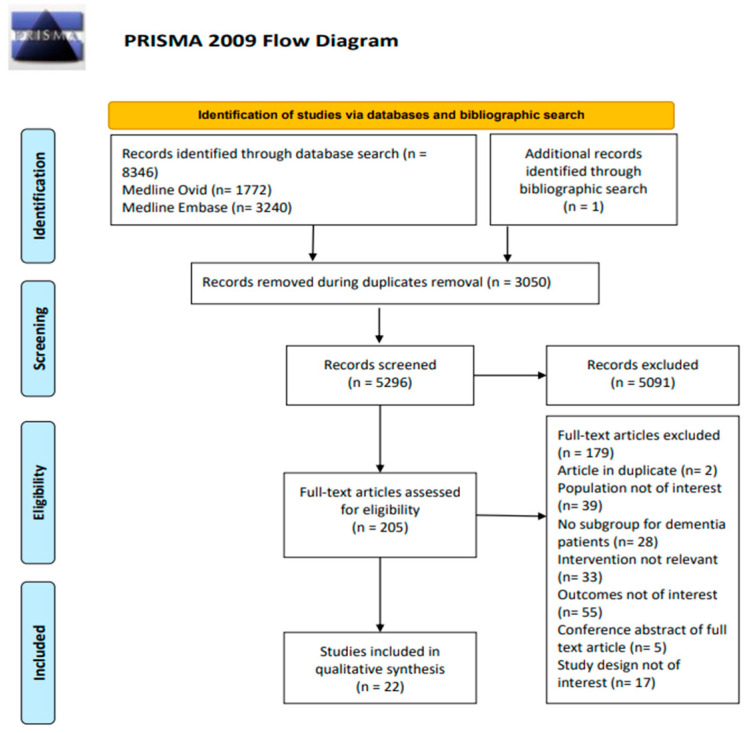
PRISMA flow diagram [24].

The initial search yielded 8346 citations; 3050 duplicates were removed. Of the remaining 5296 articles, 5091 did not meet the inclusion criteria by abstract and title. The full texts of the remaining 205 articles identified 21 articles and one conference abstract (see Figure 1).

Of the studies included, Ballard et al., 2016 [31,32], and Smeets et al., 2021 [33,34,35], published data on the same population. Gustafsson et al., 2017 [17,26,27,28], published four studies within four years commencing from 2017. A randomized controlled trial was published in 2017 and included 460 patients (intervention group = 230; control group = 230) from acute internal medicine wards and orthopedic wards [17]. Gustafsson et al. conducted three more secondary analyses using data from the RCT [26,27,28].

### 3.1. Study Characteristics

The study designs included observational pre–post studies (n = 4) [14,35,36,37], retrospective studies (n = 7) [20,38,39,40,41,42,43], prospective studies (n = 5) [44,45,46,47,48], an audit (n = 1) [49], feasibility studies (n = 2) [50,51], and randomized controlled trials (n = 3) [26,31,33]. Detailed descriptions of the included studies are summarized in Table S3.

A total of 133,024 patients were included in 22 studies. The minimum–maximum mean ages of the participants ranged from 78.33 to 87.9 years old (not reported in three studies). Out of 22 studies, 17 studies included both women and men. About 65.7% (n = 86,645) of the population in the studies were females, which is 1.9 times more than the male population in the studies (not reported in five studies).

Of the included 22 studies, 1 study each was conducted in Canada [36], the Netherlands [33], Slovenia [35], France [41], Taiwan [46], Australia [51], northern Sweden [17], Germany [43], Denmark [47], and Hong Kong [48], 5 studies were conducted in the USA [20,38,39,40,44], 3 studies were conducted in the UK [31,49,50], and 4 studies were conducted in Spain [14,37,42,45]. All the studies were published within the previous ten years.

Nine studies were conducted in long-term care facilities [31,33,36,37,42,44,45,47,50], six studies in community settings [35,38,39,40,43,51], five studies in hospital settings [14,17,37,41,48,49], and one study in all three settings and one study in both a long-term care facility and community setting [20,46].

### 3.2. Information about Interventions

Table S4 provides a summary of the interventions and their reported outcomes for each study included in the review. In terms of cognitive pharmacy services and specifically for clinical assessment (see Table S2), medication reviews were conducted by the pharmacists independently in 15 studies [17,21,36,37,38,39,40,41,42,44,47,48,49,50,51] and in collaboration with multidisciplinary teams in 6 studies [14,33,43,45,46]. One study reported a medication review conducted by a therapist [31]. The multidisciplinary teams in the six studies included a combination of a variety of healthcare professionals, such as “elderly care physicians”, nurse assistants, geriatric clinical pharmacists, physical and leisure therapists, administrators, neurologists, psychiatrists, geriatricians, primary care general practitioners, dementia specialists, nurses with expertise in dementia care, dieticians, physical therapists, occupational therapists, clinical psychologists, and social workers. Pharmacists or multidisciplinary teams identified and reported DRPs in 10 studies as part of clinical assessments in comprehensive medication management [26,39,40,41,42,44,45,48,49,51]. In eight studies, pharmacists or multidisciplinary teams also recommended appropriate interventions for DRPs identified during the medication reviews [38,39,41,42,45,47,51]. There were eight instances of pharmacists actively monitoring the outcomes of interventions and completing the essential follow-up tasks concerning the assessment part of complicated medication management [26,33,36,37,38,41,43,45,51].

Only one research study identified pharmacists as a source of drug information and counseling to people with dementia, family members, and carers [48]. In four reports for educational and advisory services to healthcare professionals, pharmacists served as a source of drug information and conducted educational sessions for other healthcare professionals [37,49,50,51].

### 3.3. Type of Outcomes Reported

**Figure 2:**
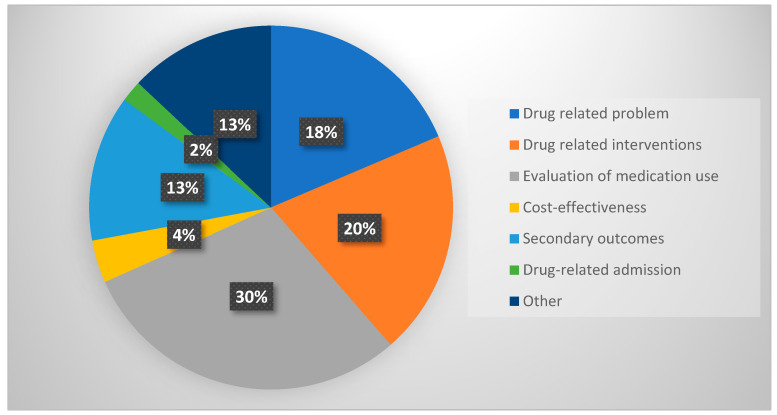
Percentages of reported outcomes by type.

Fifty-four outcomes relating to medication reviews have been reported in 22 studies. About one-fifth (10/54) of the studies have reported outcomes related to DRPs [26,39,40,41,42,44,46,48,49,51], followed by drug-related interventions (n = 11) [11,26,38,39,40,41,42,45,47,51], evaluations of medication use (n = 16) [9,21,31,33,34,35,39,40,41,42,43,44,45,46,47,49], cost-effectiveness (n = 2) [42,50], and drug-related admissions (n = 1) (see Figure 2) [45].

#### Effect of Medication Review

(A) Evaluation of medication use

The impact of medication reviews on important clinical outcomes is outlined in Table S5. Sixteen studies reported medication usage in older adults with dementia [11,21,26,27,28,29,30,31,33,36,37,39,40,41,42,43,49]. Hernandez et al. reported that 87.7% (57/65) of the population in the study was taking ≥ 5 drugs per day, and 38.5% (25/65) were on hyper-polypharmacy (taking ≥ 10 drugs per day) [45]. Almost two-thirds of the study population were prescribed antipsychotics (78.5%), followed by analgesics in 66.2%, and antidepressants in 53.9%. Nine out of ten studies reported the average number of medications per patient as ≥ 5, ranging from 6.4 to 13.3 per patient [11,26,36,37,40,44,45,49,51]. Results reported in six studies indicated a significant decrease in the average number of drugs per patient after medication reviews conducted by pharmacists independently or with multidisciplinary teams [11,26,27,28,29,30,31,36,37,49]. The intervention for one study involved a medication review conducted by a pharmacist using the medication review guidance (MRG) tool. The study was conducted among nursing home residents in Quebec. At the end of a 104-day follow-up, Wilchesky et al. found a substantial reduction in the overall number of regular drugs by 12.1% [36]. Another study reported an overall 28% decrease in the number of psychotropic drugs prescribed, with the largest decrease reported in antipsychotic use (49.66%) [37]. The intervention consisted of a review of the drugs used by the participating patients, carried out by a multidisciplinary team that involved one primary care physician and one pharmacist, as well as the nursing home doctors and nurses. At baseline, the average number of psychotropic medications administered per patient was 2.71; at one-month post-intervention, it was 1.95; and at six months, it was 2.01 (*p* ≤ 0.001 at both time points). A study conducted by Dong et al. reported the implications of Medicare Part D’s Comprehensive Medication Review (CMR) on Alzheimer’s patients’ adherence to medication [20]. The proportions of nonadherent Medicare beneficiaries in the intervention group for each prescription category decreased after they obtained a CMR, but the proportions in the comparison group grew over time. For instance, the proportion of beneficiaries in the intervention group who did not take their diabetic medications decreased from 13.1% to 9.8% in 2017. However, the percentage of nonadherent beneficiaries in the comparison group increased by 1.2%, as shown in Table S5.

(B) Drug-related problems

Ten studies reported on DRP outcomes [26,39,40,41,42,44,46,48,49,51]. Four studies defined DRPs based on established systems. For example, one study each used the Westerlund system [25], ASHP classification 1996 [52], Cipolle/Morley/Strand classification [53], and PCNE Classification V 6.2 [54], and two studies did not use any standard classification system, as shown in Table 1. The numbers of DRPs identified during medication reviews ranged from 11 to 1077. Wucherer et al. reported 1077 DRPs in 92.8% (414/446) of patients. Furthermore, the authors reported that the total number of DRPs was associated with the number of drugs taken (b = 0.07; 95% CI: 0.05–0.09; *p* < 0.001) based on a multivariate Poisson regression analysis [43]. Similar results have also been reported by another study. In one study, a multiple Cox regression model was employed to analyze the data. The results indicated that drug-related problems (DRPs) were more prevalent in certain populations. Specifically, a higher number of drugs used by individuals was associated with a greater likelihood of DRPs (odds ratio (OR): 1.255; 95% confidence interval (CI): 1.137–1.385). Additionally, populations with histories of strokes, and particularly earlier strokes, exhibited a significantly higher risk of DRPs (OR: 5.042; 95% CI: 2.032–12.509). Similarly, individuals with heart failure (OR: 2.66; 95% CI: 1.64–4.30) and diabetes mellitus (OR: 2.32; 95% CI: 1.41–3.81) were also more likely to experience DRPs [17,26,27,28].

Six studies reported outcomes on medication appropriateness [26,39,41,44,49,51]. Pharmacists’ interventions have been shown to decrease the number of PIMs used in patients after medication reviews. Pearson et al. reported a change in the mean number of PIMs in patients living with dementia from 1.5 PIMs per patient at baseline to 0.9 PIMs per patient at the 180-day follow-up after medication review [38]. In another study, the use of PIMs decreased significantly in the intervention group between admission and after medication review, from 20.3% to 14.2% (*p* = 0.002), particularly in the use of anticholinergic drugs (from 7.1% to 3.3%; *p* = 0.005) and NSAIDs, (from 3.3% to 0.9%; *p* = 0.025) [17,26,27,28]. Hernandez et al. reported a significant difference (*p* < 0.001) between the mean (SD) Medication Appropriateness Index (MAI) scores at admission and post-intervention (4 (4.6) vs. 0.5 (2.6)) [45].

PIMcog: potentially inappropriate medication for a person with cognitive impairment.

(C) Drug-related interventions

Eight studies reported the total number of proposed recommendations to the prescriber by the pharmacist or multidisciplinary team after the medication review [38,39,41,42,44,45,47,51]. In their retrospective chart review, Melville et al. present data on the identification of the number and categories of medication-related recommendations made by a geriatric clinical pharmacist in their Caring for Older adults and Caregivers at Home (COACH) Program. The geriatric clinical pharmacist proposed a total of 248 recommendations to the prescribers after the medication review [40]. The three most frequent recommendations were stopping a drug, reducing the dose, and changing to a potentially safer alternative [40]. Providers accepted 110 (44%) of the drug-related recommendations given by the pharmacist within six months of the medication review. In the Cross et al. study, pharmacy professionals made 121 deprescribing recommendations, followed by 52 on adherence and medication management, and another 88 on care-related activities, such as monitoring/investigative testing [51]. At six months, 136 of the 209 suggestions (52.1%) had either been fully or partially carried out.

## 4. Discussion

This scoping review, which examined the impact of medication reviews and interventions in older adults with dementia, found that reviews reduce polypharmacy as well as inappropriate medication use. The need for pharmacists is underlined, especially considering the issue of high-risk medicine and polypharmacy frequently seen in people with dementia [8,9,10,11,16,17]. Studies included in this scoping review suggested that the inclusion of a pharmacist care intervention had favorable results, indicating that pharmacist engagement may improve the medication management concerns in this population. The results of this scoping review are consistent with the results of McGrattan et al.’s systematic review, which highlights the positive impact on medication-related outcomes [55]. With just three papers included, this systematic review emphasizes the lack of research on medication management for persons with dementia (PWDs). Similar results are also reflected in a recently published RCT by Liu et al. on community-dwelling persons living with dementia (PLWDs) that assessed the effect of the Care Ecosystem (CE) collaborative dementia care program on the PIM use among this population. The CE resulted in significantly fewer PIMs used by PLWDs [56].

The present scoping review has determined a few clinical, practical, and scientific gaps in studies examining outcomes such as medication adherence, cost-effectiveness, and the reporting of dementia-specific core outcomes:

1. The results obtained from RCTs are the most reliable evidence to assess an intervention’s effectiveness because the randomization process can minimize the risk of bias influencing the results [57]. No RCT was conducted in the community setting for patients with dementia.
2. Only one study each was identified in this scoping review for Canada, Australia, the Netherlands, Slovenia, France, Taiwan, northern Sweden, Germany, Denmark, and Hong Kong. The studies conducted in these countries only included patients from one care setting. There is a need for more evidence for these counties in which patients are included from all types of care settings.
3. Nine studies reported data from the LTC setting, and only one, by Hernandez et al., reported DRPs in persons with dementia from the LTC setting [45]. There is a scarcity of studies reporting DRPs in persons with dementia from the LTC setting.
4. A lack of studies examining medication management and medication adherence as outcomes of medication reviews: A scoping review conducted by Hudani et al. in 2016 reported the nonadherence prevalence in older adults with CI or dementia, which ranged from 2 to 59%, which is not surprising considering the polypharmacy use, cognitive impairment, and complex medication regimens in this population [58]. Furthermore, the situation is much more difficult for individuals with CI or dementia due to various cognitive deficiencies, leading to increased nonadherence rates [58]. In this scoping review, we identified only one study that reported on medication nonadherence as an outcome of medication reviews in persons with CI and dementia [20]. Clinical practice in memory clinics includes evaluating the medication management capacity in this group. Still, it is not apparent why most of the studies did not report the effects of medication reviews on the medication management and adherence in this population. Any medication review conducted in this population should examine the medication adherence.
5. A lack of research examining the cost-effectiveness of conducting a medication review: No study in this scoping review examined the impact of medication reviews on the overall cost, such as reductions in the medication cost, hospitalization cost, medical expenses, etc. Maidment et al. have reported data on costs, such as trainer and care home staff costs [50]. The authors conducted a mixed-method feasibility study that included a comprehensive clinical medication review conducted by a specialized dementia care pharmacist. Their findings revealed that the mean cost associated with the staff time for the medication review alone was GBP 104.41 per participant. In contrast, when accounting for both the medication review and the intervention (which included training), the mean cost rose to GBP 372.80 per participant. These cost assessments provide valuable insights into the financial aspects of implementing medication review interventions in dementia care. Only one other study has reported data on the clinical, economical, and organizational dimensions of DRI in the cognitive behavioral unit. Novais et al. conducted a study from retrospective data on medication reviews in a cognitive behavioral unit (CBU) [41]. These units are designed for people with responsive behavioural abnormalities linked to Alzheimer’s disease and related dementias (ADRD). Pharmacists discovered pertinent DRPs during medication reviews and made recommendations to the patients’ physicians. A total of 543 DRPs and DRIs were recorded for patients hospitalized in the CBU. According to pharmacists, 55.2% of pharmaceutical interventions decrease the costs of care, and 16.6% increase the costs [41]. No study was found in this scoping review that reported on the cost aspect in detail or whether the medication review conducted by a pharmacist decreases the overall cost, such as reduction in the medication cost, hospitalization cost, medical expenses, etc.
6. A lack of patient and caregiver satisfaction as an outcome of medication reviews: The success of any intervention greatly relies on the patient receiving the care, the caregivers, and other healthcare professionals. The studies included in this scoping review reported no data on the satisfaction levels of patients, caregivers, or healthcare teams related to the medication review. The level of satisfaction will help the researcher to evaluate the patient, caregiver, and healthcare satisfaction and the potential acceptability of medication reviews by older adults with dementia.
7. A lack of studies reporting on quality of life: There is a scarcity of studies examining the impact of medication reviews on the quality of life in people with dementia. In this scoping review, an RCT was conducted by Ballard et al. to measure whether a review of antipsychotic medications, either alone or in conjunction with evidence-based, non-pharmacological methods, has a substantial positive impact on health-related quality of life [31]. Two DEMQOL-Proxy domains (negative emotion and appearance) significantly worsened in individuals receiving antipsychotic reviews. The DEMQOL is a 28-item self-reported tool used to assess the health-related quality of life (HRQL) of people with dementia. The caregiver fills out a 31-item examination called the DEMQOL-Proxy, which examines the patient’s cognition, adverse emotions, positive emotions, daily activities, and appearance. More studies need to be conducted to see whether the medication review increases the quality of life among older adults with dementia or not.
8. A lack of application of a dementia-specific core outcome set: The studies included in this scoping review showed variations in the measuring techniques and reported results. For instance, some studies have reported drug-related interventions without identifying DRPs, and not all studies followed up with the patients to measure the effects of the medication review. An international core outcome set for clinical trials of medication reviews in polypharmacy and multimorbid older people has been published [59]. The creation of core outcome sets for clinical trials has produced a variety of advantages, reduced the possibility of reporting bias, increased the chance of clinically meaningful results, and decreased the trial-to-trial variation in results [60]. Establishing a core outcome set for medication management interventions in primary care for individuals with dementia simplifies the research process by providing a standardized set of outcomes to evaluate the intervention’s effectiveness in this population. This approach enhances the consistency and comparability across studies, making it easier for researchers to gauge the impacts of these interventions on individuals with dementia.

### Strengths and Limitations

The robust and comprehensive search approach employed to find the range of research published globally is the main strength of this scoping review.

It is important to be aware of the limits of this scoping review. As we only considered English-language papers, language bias may have influenced it.

## 5. Conclusions

This scoping review highlights that medication reviews conducted by pharmacists independently or in collaboration with other healthcare professionals in any setting may have a positive outcome on medication use among older adults with dementia. A reduction in medication use after medication review was a key finding in this scoping review. However, this scoping review identified that studies examining quality of life, medication management, and medication adherence as outcomes of medication reviews were lacking. However, it is very difficult to draw a robust conclusion due to the variability in the reported outcomes and several limitations. The lack of standardized criteria to identify and categorize DRPs, the lack of data on comorbidities, and the lack of dementia-specific core outcomes are a few gaps that should be addressed in future research studies.

## Figures and Tables

**Table 1:** Types of drug-related problems reported.

Study	Types of Drug-Related Problems Reported
Pearson et al., 2021 [38]	2019 Beers Criteria Total of 59 PIMs identified in the 40 patients (average 1.5 PIMs/patient)
Levine et al., 2021 [39]	Unnecessary drug therapy = 1 DRP Overuse ^a^ = 6 DRPs Underuse ^b^ = 28 DRPs
Aziz et al., 2018 [49]	2015 STOPP Criteria 164 drugs prescribed
Melville et al., 2020 [40]	2012 Beers Criteria 62 (59%) patients received at least one PIM
Novais et al., 2021 [41]	Westerlund System [25] Total of 543 DRPs Non-conformity to guidelines/contra-indication = 156 (28.7%) DRPs Drug without indication = 118 DRPs Improper administration = 82 DRPs Supratherapeutic dosage = 51 DRPs Untreated indication = 40 DRPs Subtherapeutic dosage = 35 DRPs Drug monitoring = 26 DRPs Drug interaction = 17 DRPs Adverse drug reaction = 17 DRPs Failure to receive drug = 1 DRP
Hernandez et al., 2020 [45]	ASHP classification 1996 [52] Total 175 DRPs (2.97 per patient) in 90.8% of patients Actual and potential adverse drug events = 33 DRPs Medication prescribed inappropriately for a particular condition = 29 DRPs Therapeutic duplication = 18 DRPs Inappropriate dose = 17 DRPs Medication with no indication = 15 DRPs Condition for which no drug is prescribed = 14 DRPs Length = 14 DRPs Schedule = 13 DRPs Failure to receive the full benefit of prescribed therapy = 8 DRPs Actual and potential drug–drug interactions that are clinically significant = 6 DRPs Drug diseases that are clinically significant = 4 DRPs Lack of understanding of the medication = 2 DRPs Inappropriate-dose renal impairment = 1 DRPs Dosage form = 1 DRP
Cross et al., 2020 [51]	Beer’s 2015 Criteria or 2015 STOPP Criteria 25 (54.3%) patients using ≥ 1 PIM cog
Gustafsson et al., 2017 [17,26,27,28]	2015 STOPP/START Criteria 326 DRPs were identified in 153 (72.2%) patients Cipolle/Morley/Strand classification [53] Total of 310 DRPs reported in 140 (66%) patients Unnecessary drug therapy = 54 DRPs Needs additional therapy = 37 DRPs Ineffective/inappropriate drug = 54 DRPs Adverse drug reaction = 14 DRPs Too-high dosage = 44 DRPs Drug use process errors = 26 DRPs Adherence = 4 DRPs Monitoring = 13 DRPs Drug interaction = 23 DRPs
Wucherer et al., 2017 [43]	Inappropriate drugs according to the PRISCUS list reported in 105 (22.9%) patients. PCNE Classification V 6.2 [54] Total of 1077 DRPs in 414 (92.8%) patients Ineffective/inappropriate drug = 158 DRPs Adverse drug reaction = 27 DRPs Administration and compliance = 645 DRPs Drug interaction = 180 DRPs Dosage = 67 DRPs
Wong et al., 2016 [48]	Total of 11 DRPs reported

^a^ Overuse of medications refers to instances in which drugs are prescribed or taken without a clear medical necessity or indication. In the context of advanced dementia, an example of overuse would be the administration of memory-enhancing agents, which may not provide significant benefits for individuals at this stage. Similarly, the use of supplements like ginkgo or vitamin E, which lack substantial evidence for cognitive enhancement, can also be considered examples of overuse. ^b^ Underuse of medications occurs when individuals who could benefit from a particular treatment or intervention do not receive it. In the case of dementia, underuse was identified in situations in which individuals met specific criteria but were not receiving pharmacotherapy. This included individuals with Montreal Cognitive Assessment (MoCA) scores of 25 or lower who were designated as having dementia based on the study’s criteria. However, individuals with advanced dementia (MoCA scores below 10) were excluded from consideration for medication, as the potential benefits in this group were deemed to be limited.

## Supplementary Materials

The following supporting information can be downloaded at https://www.mdpi.com/article/10.3390/pharmacy11050168/s1, Table S1. Search strategy; Table S2. Types of care settings, pharmacist care interventions, DRPs, and drug-related interventions (DRIs); Table S3. Characteristics of included studies (n = 22) of the scoping review; Table S4. Summary of interventions with reported outcomes; Table S5. Overview of medication review and important clinical outcomes reported.

## References

[1] Hanjani L.S., Long D., Peel N.M., Peeters G., Freeman C.R., Hubbard R.E. (2019). Interventions to optimise prescribing in older people with dementia: A systematic review. Drugs Aging.

[2] World Health Organization Dementia. http://www.who.int/mediacentre/factsheets/fs362/en/.

[3] World Health Organization. https://www.who.int/news/item/07-12-2017-dementia-number-of-people-affected-to-triple-in-next-30-years.

[4] Dementia in Canada, Including Alzheimer’s Disease. https://www.canada.ca/en/public-health/services/publications/diseases-conditions/dementia-highlights-canadian-chronic-disease-surveillance.html.

[5] Chung S.D., Liu S.P., Sheu J.J., Lin C.C., Lin H.C., Chen C.H. (2014). Increased healthcare service utilizations for patients with dementia: A population-based study. PLoS ONE.

[6] Alzheimer Society Canada Dementia Numbers in Canada. https://alzheimer.ca/en/about-dementia/what-dementia/dementia-numbers-canada#ref5.

[7] Schubert C.C., Boustani M., Callahan C.M., Perkins A.J., Carney C.P., Fox C., Unverzagt F., Hui S., Hendrie H.C. (2006). Comorbidity profile of dementia patients in primary care: Are they sicker?. J. Am. Geriatr. Soc..

[8] Clague F., Mercer S.W., McLean G., Reynish E., Guthrie B. (2017). Comorbidity and polypharmacy in people with dementia: Insights from a large, population-based cross-sectional analysis of primary care data. Age Ageing.

[9] Growdon M.E., Gan S., Yaffe K., Steinman M.A. (2021). Polypharmacy among older adults with dementia compared with those without dementia in the United States. J. Am. Geriatr. Soc..

[10] Lau D.T., Mercaldo N.D., Harris A.T., Trittschuh E., Shega J., Weintraub S. (2010). Polypharmacy and potentially inappropriate medication use among community-dwelling elders with dementia. Alzheimer Dis. Assoc. Disord..

[11] Johnell K. (2015). Inappropriate drug use in people with cognitive impairment and dementia: A systematic review. Curr. Clin. Pharmacol..

[12] Ramsey C.M., Gnjidic D., Agogo G.O., Allore H., Moga D. (2018). Longitudinal patterns of potentially inappropriate medication use following incident dementia diagnosis. Alzheimer’s Dement. Transl. Res. Clin. Interv..

[13] Renom-Guiteras A., Thürmann P.A., Miralles R., Klaaßen-Mielke R., Thiem U., Stephan A., Bleijlevens M.H., Jolley D., Leino-Kilpi H., Rahm Hallberg I. (2018). Potentially inappropriate medication among people with dementia in eight European countries. Age Ageing.

[14] Brunet N.M., Sevilla-Sánchez D., Novellas J.A., Jané C.C., Gómez-Batiste X., McIntosh J., Panicot J.E. (2014). Optimizing drug therapy in patients with advanced dementia: A patient-centered approach. Eur. Geriatr. Med..

[15] Smith D., Lovell J., Weller C., Kennedy B., Winbolt M., Young C., Ibrahim J. (2017). A systematic review of medication non-adherence in persons with dementia or cognitive impairment. PLoS ONE.

[16] Eshetie T.C., Nguyen T.A., Gillam M.H., Ellett L.M.K. (2018). A narrative review of problems with medicines use in people with dementia. Expert Opin. Drug. Saf..

[17] Pfister B., Jonsson J., Gustafsson M. (2017). Drug-related problems and medication reviews among old people with dementia. BMC Pharmacol. Toxicol..

[18] Van Spall H.G., Toren A., Kiss A., Fowler R.A. (2007). Eligibility criteria of randomized controlled trials published in high-impact general medical journals: A systematic sampling review. JAMA.

[19] Reeve E., Trenaman S.C., Rockwood K., Hilmer S.N. (2017). Pharmacokinetic and pharmacodynamic alterations in older people with dementia. Expert Opin. Drug. Metab. Toxicol..

[20] Dong X., Tsang C.C.S., Zhao S., Browning J.A., Wan J.Y., Chisholm-Burns M.A., Finch C.K., Tsao J.W., Hines L.E., Wang J. (2021). Effects of the Medicare Part D comprehensive medication review on medication adherence among patients with Alzheimer’s disease. Curr. Med. Res. Opin..

[21] PCNE Working Group on Medication Review. https://www.pcne.org/working-groups/1/medication-review.

[22] Krska J., Cromarty J.A., Arris F., Jamieson D., Hansford D., Duffus P.R., Downie G., Seymour D.G. (2001). Pharmacist-led medication review in patients over 65: A randomized, controlled trial in primary care. Age Ageing.

[23] Arksey H., O’Malley L. (2005). Scoping studies: Towards a methodological framework. Int. J. Soc. Res. Methodol..

[24] Tricco A.C., Lillie E., Zarin W., O’Brien K.K., Colquhoun H., Levac D., Moher D., Peters M.D., Horsley T., Weeks L. (2018). PRISMA extension for scoping reviews (PRISMA-ScR): Checklist and explanation. Ann. Intern. Med..

[25] van Mil J.F., Westerlund L.T., Hersberger K.E., Schaefer M.A. (2004). Drug-related problem classification systems. Ann. Pharmacother..

[26] Gustafsson M., Sjölander M., Pfister B., Jonsson J., Schneede J., Lövheim H. (2017). Pharmacist participation in hospital ward teams and hospital readmission rates among people with dementia: A randomized controlled trial. Eur. J. Clin. Pharmacol..

[27] Gustafsson M., Sjölander M., Pfister B., Schneede J., Lövheim H. (2018). Effects of Pharmacists’ Interventions on Inappropriate Drug Use and Drug-Related Readmissions in People with Dementia—A Secondary Analysis of a Randomized Controlled Trial. Pharmacy.

[28] Abramsson L., Gustafsson M. (2020). Prevalence of drug-related problems using STOPP/START and medication reviews in elderly patients with dementia. Res. Soc. Adm. Pharm..

[29] Sanford A.M., Orrell M., Tolson D., Abbatecola A.M., Arai H., Bauer J.M., Cruz-Jentoft A.J., Dong B., Ga H., Goel A. (2015). An international definition for “nursing home”. J. Am. Med. Dir. Assoc..

[30] Lee C., Ivo J., Carter C., Faisal S., Shao Y.W., Patel T. (2021). Pharmacist interventions for persons with intellectual disabilities: A scoping review. Res. Soc. Adm. Pharm..

[31] Ballard C., Orrell M., Yongzhong S., Moniz-Cook E., Stafford J., Whittaker R., Woods B., Corbett A., Garrod L., Khan Z. (2016). Impact of antipsychotic review and nonpharmacological intervention on antipsychotic use, neuropsychiatric symptoms, and mortality in people with dementia living in nursing homes: A factorial cluster-randomized controlled trial by the well-being and health for people with dementia (WHELD) program. Am. J. Psychiatry.

[32] Ballard C., Orrell M., Sun Y., Moniz-Cook E., Stafford J., Whitaker R., Woods B., Corbett A., Banerjee S., Testad I. (2017). Impact of antipsychotic review and non-pharmacological intervention on health-related quality of life in people with dementia living in care homes: WHELD—A factorial cluster randomised controlled trial. Int. J. Geriatr. Psychiatry.

[33] Smeets C.H.W., Smalbrugge M., Koopmans R.T.C.M., Nelissen-Vrancken M.H.J.M.G., Van Der Spek K., Teerenstra S., Gerritsen D.L., Zuidema S.U. (2021). Can the PROPER intervention reduce psychotropic drug prescription in nursing home residents with dementia? Results of a cluster-randomized controlled trial. Int. Psychogeriatr..

[34] van der Spek K., Koopmans R.T., Smalbrugge M., Nelissen-Vrancken M.H., Wetzels R.B., Smeets C.H., De Vries E., Teerenstra S., Zuidema S.U., Gerritsen D.L. (2018). The effect of biannual medication reviews on the appropriateness of psychotropic drug use for neuropsychiatric symptoms in patients with dementia: A randomised controlled trial. Age Ageing.

[35] Stuhec M., Lah L. (2021). Clinical pharmacist interventions in elderly patients with mental disorders in primary care focused on psychotropics: A retrospective pre–post observational study. Ther. Adv. Psychopharmacol..

[36] Wilchesky M., Mueller G., Morin M., Marcotte M., Voyer P., Aubin M., Carmichael P.H., Champoux N., Monette J., Giguère A. (2018). The OptimaMed intervention to reduce inappropriate medications in nursing home residents with severe dementia: Results from a quasi-experimental feasibility pilot study. BMC Geriatr..

[37] Mesquida M.M., Casas M.T., Sisó A.F., Muñoz I.G., Vian Ó.H., Monserrat P.T. (2019). Consensus and evidence-based medication review to optimize and potentially reduce psychotropic drug prescription in institutionalized dementia patients. BMC Geriatr..

[38] Pearson S.M., Osbaugh N.A., Linnebur S.A., Fixen D.R., Brungardt A., Marcus A.M., Lum H.D. (2021). Implementation of Pharmacist Reviews to Screen for Potentially Inappropriate Medications in Patients with Cognitive Impairment. Sr. Care Pharm..

[39] Levine A.M., Emonds E.E., Smith M.A., Rickles N.M., Kuchel G.A., Steffens D.C., Ohlheiser A., Fortinsky R.H. (2021). Pharmacist identification of medication therapy problems involving cognition among older adults followed by a home-based care team. Drugs Aging.

[40] Melville B.L., Bailey J., Moss J., Bryan W., Davagnino J., Twersky J., Pepin M. (2020). Description of pharmacist recommendations in the Caring for Older Adults and Caregivers at Home (COACH) Program. Sr. Care Pharm..

[41] Novais T., Maldonado F., Grail M., Krolak-Salmon P., Mouchoux C. (2021). Clinical, economic, and organizational impact of pharmacists’ interventions in a cognitive-behavioral unit in France. Int. J. Clin. Pharm..

[42] Weeks W.B., Mishra M.K., Curto D., Petersen C.L., Cano P., Hswen Y., Serra S.V., Elwyn G., Godfrey M.M., Soro P.S. (2019). Comparing three methods for reducing psychotropic use in older demented Spanish care home residents. J. Am. Geriatr. Soc..

[43] Wucherer D., Thyrian J.R., Eichler T., Hertel J., Kilimann I., Richter S., Michalowsky B., Zwingmann I., Dreier-Wolfgramm A., Ritter C.A. (2017). Drug-related problems in community-dwelling primary care patients screened positive for dementia. Int. Psychogeriatr..

[44] Bach L.L., Lazzaretto D.L., Young C.F., Lofholm P.W. (2017). Improving nursing home compliance via revised antipsychotic use survey tool. Consult. Pharm..

[45] Hernandez M., Mestres C., Junyent J., Costa-Tutusaus L., Modamio P., Lastra C.F., Mariño E.L. (2020). Effects of a multifaceted intervention in psychogeriatric patients: One-year prospective study. Eur. J. Hosp. Pharm..

[46] Liang C.K., Chou M.Y., Chen L.Y., Wang K.Y., Lin S.Y., Chen L.K., Lin Y.T., Liu T.Y., Loh C.H. (2017). Delaying cognitive and physical decline through multidomain interventions for residents with mild-to-moderate dementia in dementia care units in Taiwan: A prospective cohort study. Geriatr. Gerontol. Int..

[47] Tang M.M., Wollsen M.G., Aagaard L. (2016). Pain monitoring and medication assessment in elderly nursing home residents with dementia. J. Res. Pharm. Pract..

[48] Wong Y.L., Cheung K.L., Chan C.C., Yung C.Y. (2016). P3-329: Pharmacist-Managed Medication Review in a Novel Multidisciplinary Care Model for Elderly with Dementia. Alzheimer’s Dement..

[49] Aziz V.M., Hill N., Kumar S. (2018). Completed audit cycle to explore the use of the STOPP/START toolkit to optimise medication in psychiatric in-patients with dementia. BJPsych. Bull..

[50] Maidment I.D., Barton G., Campbell N., Shaw R., Seare N., Fox C., Iliffe S., Randle E., Hilton A., Brown G. (2020). MEDREV (pharmacy-health psychology intervention in people living with dementia with behaviour that challenges): The feasibility of measuring clinical outcomes and costs of the intervention. BMC Health Serv..

[51] Cross A.J., George J., Woodward M.C., Le V.J., Elliott R.A. (2020). Deprescribing potentially inappropriate medications in memory clinic patients (DePIMM): A feasibility study. Res. Soc. Adm. Pharm..

[52] American Society of Health-System Pharmacists (1996). ASHP guidelines on a standardized method for pharmaceutical care. Am. J. Health-Syst. Pharm..

[53] Cipolle R., Strand L., Morely P. (1998). Pharmaceutical Care Practice.

[54] Pharmaceutical Care Network Europe Foundation (2010). The PCNE Classification V 6.2—Classification for Drug Related Problems. http://www.pcne.org/upload/files/11_PCNE_classification_V6-2.pdf.

[55] McGrattan M., Ryan C., Barry H.E., Hughes C.M. (2017). Interventions to improve medicines management for people with dementia: A systematic review. Drugs Aging.

[56] Liu A.K., Possin K.L., Cook K.M., Lynch S., Dulaney S., Merrilees J.J., Braley T., Kiekhofer R.E., Bonasera S.J., Allen I.E. (2022). Effect of collaborative dementia care on potentially inappropriate medication use: Outcomes from the Care Ecosystem randomized clinical trial. Alzheimer’s Dement..

[57] Akobeng A.K. (2005). Understanding randomised controlled trials. Arch. Dis. Childh..

[58] Hudani Z.K., Rojas-Fernandez C.H. (2016). A scoping review on medication adherence in older patients with cognitive impairment or dementia. Res. Soc. Adm. Pharm..

[59] Beuscart J.B., Knol W., Cullinan S., Schneider C., Dalleur O., Boland B., Thevelin S., Jansen P.A., O’Mahony D., Rodondi N. (2018). International core outcome set for clinical trials of medication review in multi-morbid older patients with polypharmacy. BMC Med..

[60] McGrattan M., Barry H.E., Ryan C., Cooper J.A., Passmore A.P., Robinson A.L., Molloy G.J., Darcy C.M., Buchanan H., Hughes C.M. (2019). The development of a core outcome set for medicines management interventions for people with dementia in primary care. Age Ageing.

