# Supplementary material for: Medication Reviews and Clinical Outcomes in Persons with Dementia: A Scoping Review
Source: Pharmacy (Basel). 2023 Oct 20;11(5):168. doi: 10.3390/pharmacy11050168 (PMC10609910; doi:10.3390/pharmacy11050168)
Supplement: Supplementary file 1 [file pharmacy-11-00168-s001.zip › pharmacy-2572110-supplementary.pdf]

Table S1. Search strategy.

OVID Medline

#	Search strategy	Medline hits
1	exp Aged/	3372444
2	((older$ or elderly or geriatric) adj2 (adult$ or people$ or patient$ or in patient$ or in-patient$ or inpatient$)).ti,ab,kw	291927
3	(aged or frail elderly or (health services adj3 aged) or community dwelling older adults).ti,ab,kw	648093
4	or/1-3	3857586
5	exp Dementia/ or exp Alzheimer Disease/ or exp Dementia, Vascular/ or exp Frontotemporal Dementia/	187161
6	exp Cognition Disorders/ or exp Cognition/ or exp Memory Disorders/	297449
7	exp Lewy Body Disease/	3864
8	exp Korsakoff Syndrome/	545
9	(dement$ or (alzheimer or (lewy adj2 bod$) adj2 diseas$) or (chronic adj2 cerebrovascular)).ti,ab,kw	142515
10	(organic brain disease or organic brain syndrome).ti,ab,kw	796
11	(cerebr$ adj2 (deteriorat$ or insufficien$) or binswanger$ or (pick$ disease)).ti,ab,kw	5962
12	(behavio?r$ adj2 (modif$ or chang$ or improv$)).ti,ab,kw	74553
13	or/5-12	555768
14	4 and 13	175244
15	exp Medication Reconciliation/ or exp Medication Adherence/	25488
16	((prescription$ or prescribing or medication$ or medicine$ or drug therapy or pharmac$ or drug regime$ or drug therap$ or pharmaceutical care or dosage$ or dose$) adj3 (review$ or assess$ or audit$ or monitor$ or reconcil$ or manag$ or monitor$ or plan or record or adher$ or concord$)).ti,ab,kw	113529
17	15 or 16	128535
18	14 and 17	1857
19	Limit 18 to English language	1772

OVID Embase

#	search strategy	Medline hits
1	exp Aged/	3316437

2	((older$ or elderly or geriatric) adj2 (adult$ or people$ or patient$ or in patient$ or in-patient$ or inpatient$)).ti,ab,kw	418145
3	(aged or frail elderly or (health services adj3 aged) or community dwelling older adults).ti,ab,kw	896078
4	or/1-3	4036298
5	exp Dementia/ or exp Alzheimer Disease/ or exp multiinfarct dementia/ or exp Frontotemporal Dementia/ or exp frontal variant frontotemporal dementia/ or Pick presenile dementia/ or semantic dementia/ or senile dementia/ or presenile dementia/	399376
6	exp cognitive defect/ or exp Memory Disorders/	598959
7	exp diffuse Lewy body disease/	10136
8	exp Korsakoff psychosis/	1550
9	(dement$ or (alzheimer or (lewy adj2 bod$) adj2 diseas$) or (chronic adj2 cerebrovascular)).ti,ab,kw	210743
10	(organic brain disease or organic brain syndrome).ti,ab,kw	1040
11	(cerebr$ adj2 (deteriorat$ or insufficien$) or binswanger$ or (pick$ disease)).ti,ab,kw	7535
12	(behavio?r$ adj2 (modif$ or chang$ or improv$)).ti,ab,kw	96104
13	or/5-12	719253
14	4 and 13	201783
15	exp Medication Reconciliation/ or exp Medication compliance/	51136
16	((prescription$ or prescribing or medication$ or medicine$ or drug therapy or pharmac$ or drug regime$ or drug therap$ or pharmaceutical care or dosage$ or dose$) adj3 (review$ or assess$ or audit$ or monitor$ or reconcil$ or manag$ or monitor$ or plan or record or adher$ or concord$)).ti,ab,kw	188256
17	15 or 16	214686
18	14 and 17	3329
19	Limit 18 to English language	3240

SCOPUS

#	search strategy	Scopus
#1	TITLE-ABS-KEY ((*older** OR *"elderly"* OR *"geriatric"*) W/2 (*adult** OR *people ** OR *patient** OR *"in patient"* OR *"in-patient"* OR *"inpatient"*))	416,837
#2	TITLE-ABS-KEY (*"aged"* OR *"frail elderly"* OR (*"health services"* W/3 *"aged"*) OR *"community dwelling older adults"*)	5,766,189

#3	#1 OR #2	5,887,136
#4	TITLE-ABS-KEY (*"Dementia"* OR *"Alzheimer Disease"* OR "Alzheimer disorder" OR *"multiinfarct dementia"* OR *"Frontotemporal Dementia"* OR *"frontal variant frontotemporal dementia"* OR *"Pick presenile dementia"* OR *"semantic dementia"* OR *"senile dementia"* OR *"presenile dementia"*)	371,317
#5	TITLE-ABS-KEY (*"Cognition disorder"* OR *"Cognition"* OR *"Memory disorder"*)	448,722
#6	TITLE-ABS-KEY (*"Lewy body disease"*)	8,237
#7	TITLE-ABS-KEY (*"Korsakoff Syndrome"* OR *"Korsakoff psychosis"*)	2,703
#8	TITLE-ABS-KEY (*"Chronic"* W/2 *"cerebrovascular"*)	1,306
#9	TITLE-ABS-KEY (*"organic brain disease"* OR *"organic brain syndrome"*)	3,587
#10	TITLE-ABS-KEY (*cerebr** W/2 (*deteriorat** OR *insufficien**) OR *binswanger** OR *pick** AND *disease*)	2,446
#11	TITLE-ABS-KEY (*behavio?r** W/2 (*modif** OR *chang** OR *improv**))	61,824
#12	#4 OR #5 OR #6 OR #7 OR #8 OR #9 OR #10 OR #11	819,232
#13	#3 AND #12	249,393
#14	TITLE-ABS-KEY ((*prescription** OR *"prescribing"* OR *medication** OR *medicin** OR *"drug therapy"*) W/3 (*review** OR *assess** OR *audit** OR *monitor**))	48,267
#15	TITLE-ABS-KEY ((*prescription** OR *"prescribing"* OR *medication** OR *medicin** OR *"drug therapy"*) W/3 (*reconcil** OR *manag** OR *monitor** OR *plan*))	45,140
#16	TITLE-ABS-KEY ((*prescription** OR *"prescribing"* OR *medication** OR *medicin** OR *"drug therapy"*) W/3 (*"record"* OR *adher** OR *concord**))	41,616
#17	TITLE-ABS-KEY ((*pharmac** OR *"drug regimen"* OR *"pharmaceutical care"* OR *dosage** OR *dose**) W/3 (*review** OR *assess** OR *audit** OR *monitor**))	75,683
#18	TITLE-ABS-KEY ((*pharmac** OR *"drug regimen"* OR *"pharmaceutical care"* OR *dosage** OR *dose**) W/3 (*reconcil** OR *manag** OR *monitor** OR *plan*))	46,372
#19	TITLE-ABS-KEY ((*pharmac** OR *"drug regimen"* OR *"pharmaceutical care"* OR *dosage** OR *dose**) W/3 (*"record"* OR *adher** OR *concord**))	8,372
#20	#14 OR #15 OR #16 OR #17 OR #18 OR #19	216,220
#21	#13 AND #20	3,470
#22	LIMIT-TO (LANGUAGE , "English")	3,333

Table S2. Definitions of types of care settings, pharmacist care interventions, drug related problems (DRPs) and drug related interventions (DRIs).

	**Care settings**	**Description**
Type of care settings	Community	Patients receiving primary healthcare services outside of a designated accommodation facility while living independently or with family members [30]
	Hospital	Facility that is distinguishable from a long-term care facility due to well established differences in the type and duration of specialist medical treatment [30]
	Long-term care facility	Patients receiving primary healthcare services at the nursing homes/skilled nursing facilities/assisted living/residential living homes [29]
	**Class**	**Description of main categories**
Type of Pharmacist Care Interventions	Cognitive Pharmacy Services (CPS)	Comprehensive Medication Management<br>• With medications prescribed from another health care professional.<br>Subdivided into 3 steps:<br>1) Clinical assessment [30,35,38,40]<br>• Collect general medication history and other key clinical information<br>• Conduct medication review<br>• Identify drug related problems (DRPs)<br>2) Care Plan Creation and Implementation [38,40]<br>• Recommend interventions and solutions for DRPs<br>3) Evaluation [30,38,40]<br>• Follow-up and monitor results
	Educational and Advisory Services [38,59]	Secondary Patient Care Services<br>• Perform additional patient care services, e.g., administer drugs via injection<br>Management of Minor Conditions<br>• Assessment and diagnosis, triage/referral, treatment, monitor and follow-up<br>Patients, Family members, and Caregivers<br>• Provide drug counselling services<br>Health Care Professionals

		• Provide advice and explanations on drug information and rationale for medication use<br>Other health care workers e.g., staff workers at facilities<br>• Provide advice and explanations on drug information and rationale for medication use
	**Source**	**Type of DRPs**
Type of DRPs	Westerlund classification system [25], ASHP classification 1996 [52], Cipolle/Morley/Strand classification [53], PCNE Classification V 6.2 [54]	• Non-conformity to guidelines / contra-indication<br>• Drug without indication<br>• Improper administration<br>• Supratherapeutic dosage<br>• Untreated indication<br>• Subtherapeutic dosage<br>• Unnecessary Drug Therapy<br>• Needs Additional Therapy<br>• Ineffective/Inappropriate drug<br>• Too High Dosage<br>• Drug use process errors: DRPs that occur due to inappropriate administration by a care provider<br>• Drug monitoring<br>• Drug interaction<br>• Adverse drug reaction<br>• Failure to receive drug<br>• Length<br>• Schedule<br>• Failure to receive the full benefit of prescribed therapy<br>• Drug-disease that are clinically significant<br>• Lack of understanding of the medication<br>• Inappropriate dose renal impairment<br>• Dosage form
	**Source**	**Type of DRIs**
Type of DRIs	Pharmaceutical Care Network Europe Classification for Drug related problems [21]	• No intervention<br>• At prescriber level<br>• At patient level<br>• At drug level

		• Other intervention (e.g., side effect monitoring, health, and medicine monitoring)

Table S3. Characteristics of included studies (n= 22) of the scoping review.

Study	Study characteristics		Participant characteristics							
	Design; follow up; medication review conducted by	Type of care setting; single center/multicenter	Sample size (N=)	Study population	Age	Sex	Classification of dementia	Secondary conditions	Evaluation of medication use	Other
**Canada**										
1. Wilchesky et al., 2018 [36]	Observational pre-post study; 104 days; Clinical pharmacist	LTC; Multicenter	44	Nursing home residents 65 years of age or older with diagnosis of severe dementia	86.9 (6.9) (mean (SD))	70.5% F 29.5% M	NR	7.45 (2.46) (mean (SD)) Charlson Comorbidity Score	7.86 (3.78) (mean (SD)) Number of Medication per patient	Level of Agitation and Pain
**United States**										
2. Dong et al., 2021 [20]	Retrospective study; NR; Clinical pharmacist	Community, LTC, Hospital; Multicenter	129,820 (Intervention= 32,455; comparison group= 97,365)	Aged 65 years or older had AD	79.04 (7.37) (mean (SD))-intervention 78.93 (7.86) (mean (SD))-comparison	34.18% M 65.82% F intervention 34.18% M 65.82% F comparison	NR	NR	NR	Proportions of nonadherent beneficiaries
3. Pearson et	Retrospective study; NR;	Community (Academic geriatric	40	Community-dwelling patients	82.4 (67-98)	37.5% M 62.5% F	NR	NR	Donepezil Monotherapy (n= 3)	

al., 2021 [38]	Clinical pharmacist	primary care clinics); Multicenter		with dementia	(mean (range))				Donepezil + Memantine (n= 4)<br>Rivastigmine + Memantine (n= 2)<br>Galantamine + Memantine (n= 1)<br>Memantine Monotherapy (n= 7)	
4. Bach et al., 2017 [44]	Prospective study; NR; Clinical pharmacist	LTC (Nursing homes); Multicenter	20	Nursing home residents diagnosed with dementia	87.1 (7.9) (mean (SD))	90% F<br>10% M	NR	NR	13.3 (5.5) (mean (SD)) number of medications<br>Olanzapine 25%<br>Quetiapine 60%<br>Risperidone 15%	
5. Levine et al., 2021 [39]	Retrospective study; NR; Clinical pharmacist	Community (Living home); Single center	29	Older adults aged ≥ 65 years, living at home with dementia	78.9 (7.2) (mean (SD))	48.3% F<br>51.7% M	NR	3.21 (1.5) Average comorbidities per patient	8.3 (3.9) (mean (SD)) number of medications<br>Memory agents 41.7%<br>Acetylcholinesterase inhibitors 43.3%<br>NMDA antagonist 38.95%<br>Central nervous system agents 10.7%<br>Antidepressants 9.6%<br>Anxiolytics 4.2%<br>Anticonvulsants 26.7%<br>Antiparkinsonian agents 28.6%<br>Anticholinergic agents 10%	
6. Melville et al., 2020 [40]	Retrospective study; NR; Geriatric clinical pharmacist	Community (Tertiary care Veterans Affairs health care	104	Older Adults with dementia attending Outpatients in a tertiary	81 (65-99) (mean (range))	4% F<br>96% M	NR	NR	NR	

		system Outpatients); Single center		care Veterans Affairs health care system						
**United Kingdom**										
6. Aziz et al., 2018 [49]	Audit study; NR; Consultant Pharmacist	Hospital (Cwm Taf UHB); Multicenter	58 first audit 47 re-audit	Psychiatric in-patients with dementia	78.33 (2.74) (mean (SD)) first audit 78.72 (3.11) re-audit	53.5% F 46.5% M first audit 63% F 37% M Re-audit	Alzheimer's dementia (n= 18) Vascular dementia (n= 21) Dementia with Lewy bodies (n= 5) Mixed Alzheimer's/vascular dementia (n= 7) Other dementia (n= 6)	6.23 (1.52) Average comorbidities per patient- first audit 5.73 (1.02) re-audit	10.88 (1.27) Average number of prescriptions per patient- first audit 10.15 (0.58) re-audit	
7. Ballard et al., 2016 [31] (primary study)	Randomized controlled trial; NR; Therapist	LTC (Nursing Care homes); Multicenter	277 (Residents on anti-psychoti	People with dementia living in nursing homes	85.26 (7.02) (mean (SD))	74% F 26% M	NR	NR	NR	Cohen-Mansfield Agitation Inventory score, Neuropsychiatri

Ballard et al., 2017 [32] (secondary study)			c review 146; Residents not on anti-psychotic review= 131)							c Inventory score, quality of life
8. Maidment et al., 2020 [50]	Feasibility study; 6 months; Specialist dementia care pharmacist	LTC (Residents in care homes); Multicenter	29	People living with moderate to severe dementia	83.6 (9.3) (mean (SD)) 66- 100 (range)	62.1% F 37.9% M	NR	NR	medication involved in medication reviews, citalopram (n= 6) Sertraline (n= 4) Mirtazapine (n= 4) Antihistamines (n= 3) Trimipramine (n= 1) Amisulpride (n= 1)	Neuropsychiatric Inventory score, quality of life
**Netherlands**										
9. Smeets et al., 2021 [33] (primary study)	Randomized controlled trial; 18 months; Elderly care physician, pharmacist, nurse (assistant)	LTC; Multicenter	222	Nursing home residents living in the participating dementia special care units (DSCUs)	84 (7.4) (mean (SD)) 55–99 (range)	78% F 22% M	Alzheimer's dementia 41% Vascular dementia 12% Mixed Alzheimer's/vascular dementia 10% Other dementia 37%		Any antipsychotic, antidepressant, hypnotic, and/or anxiolytic:- 48% Antipsychotics25% Antidepressants 25% Hypnotics (14%) Anxiolytics (14%)	Cohen-Mansfield Agitation Inventory score, Neuropsychiatric Inventory score
Van Der Spek et al., 2018 [34] (secondary study)										
**Slovenia**										

10. Stuhec et al., 2021 [35]	Observational pre–post study; NR; Clinical pharmacist	Community; Multicenter	19	Elderly patients aged 65 years or above diagnosed with dementia	NR	NR	NR	NR	NR	
**France**										
12. Novais et al., 2021 [41]	Retrospective study; NR; Senior pharmacists or resident pharmacists	Hospital (Cognitive-behavioral unit); Single center	543	Elderly patients admitted in a cognitive-behavioral unit with Alzheimer's disease and Related Dementia (ADRD))	79.0 (9.5) (mean (SD))	NR	NR	NR	NR	Economic, and organizational impact
**Spain**										
13. Weeks et al., 2019 [42]	Retrospective study; 4 weeks; Carers, nursing staff, physicians, physical & leisure therapists, and administrators	LTC (Nursing homes); Multicenter	606		NR	NR	NR	NR	NR	

14. Massot et al., 2019 [37]	Prospective, observational pre-post study; 6 months; Neurologist, a psychiatrist, a geriatrician, 2 primary care general practitioners and 4 pharmacists,	LTC (Nursing homes associated with a single primary care team); Multicenter	240	Institutionalized patients diagnosed with dementia	87.9 (6.8) (mean (SD))	75% F 25% M	NR	NR	2.71 (1.47) average number psychotropic drugs/patient	
15. Hernandez et al., 2020 [45]	Prospective study; NR; Pharmacist and a geriatrician	LTC (long-term care psychogeriatric unit (21 beds) in an intermediate care hospital); Single center	65	Patients with dementia admitted to control behavioural and psychological symptoms	84.9 (6.7) (mean (SD))	60% F 40% M	Alzheimer's dementia 30.8% Vascular dementia 7.7% Dementia with Lewy bodies 7.7% Mixed Alzheimer's/vascular dementia 4.6%	Diseases of the circulatory system 83.1% Endocrine, nutritional, and metabolic diseases 60% genitourinary system 32.3% musculoskeletal system and connective tissue 29.2% nervous system 27.7% Neoplasms 16.9% Injury, poisoning, and certain other consequences 26.2%	9.0 (3.1) average number psychotropic drugs/patient Antipsychotics 78.5% hypnotics and sedatives)/anxiolytics 47.7% antidepressants 53.9% analgesics 66.2% anti-dementia drugs 30.9% antiepileptic drugs 12.3% anti-Parkinson drugs 4.6%	Anticholinergic burden

							Other dementi a 6.2%	digestive system 23.1%<br>eye and adnexa 16.9%<br>blood and blood-forming organs 15.4%<br>Mental and behavioural disorders 15.4%<br>respiratory system 12.3%<br>skin and subcutaneous tissue 1.5%		
16. Molist et al., 2014 [14]	Observational pre-post study; NR; Two geriatricians and a clinical pharmacist	Hospital (advanced dementia admitted to acute geriatric unit); Single center	73	Patients with advanced dementia	86.1 (5.73) (mean (SD)) 72–100 (range)	79.45% F 20.55% M	NR	Trauma 35.61%<br>Infection 36.98%<br>Respiratory infections 44.34%<br>Urinary tract infections 33.26%<br>Cardiovascular disease 20.54%	7.27 average of medications prior to hospitalization	
**Taiwan**										
17. Liang et al., 2017 [46]	Prospective study; 12 months; Dementia specialist, a special nurse with expertise in dementia care, a pharmacist, a	LTC and Community (intervention in Jia-Li Veterans Home and usual care model in the community (Memory	61	Participants aged 65 years and older with mild-to-moderate dementia	85.8 (5.6) (mean (SD))	NR	NR	NR	Use of anti-dementia drug included acetylcholinesterase inhibitor and memantine 88.5%	Delaying cognitive and physical decline, and improvement or prevention of geriatric syndromes during 1-year follow up

	dietician, a physical therapist, an occupational therapist, a clinical psychologist and social workers	clinic)); Multicenter								
**Australia**										
18. Cross et al., 2020 [51]	Pre- and post-intervention feasibility study; 6 months; Two consultant pharmacists	Community (outpatient memory clinics); Single center	50	Patients attending the memory clinics	80.5 (71.5-85.0) (median (IQR))	36% F 64% M	Alzheimer's dementia 16% Mixed dementia 14% Mild cognitive impairment 26% Not confirmed diagnosis 26%	4.94 (1.89) (mean (SD)) Charlson comorbidity index	11 (8-13.25) (median (IQR)) Median number of medications at home visit	Quality of life (EQ-5D), tool for adherence behaviour screening Adherence
**Northern Sweden**										
19. Gustafsson et al., 2017 [26] (primary study)	Randomized controlled trial; 6 months; Three	Hospital (Patients admitted to acute internal medicine	212	65 years or older and had dementia	83.1 (6.6) (mean (SD))	63% F 37% M	Alzheimer's dementia 30% Vascular	Heart failure 34% Hypertension 55% Cardiac arrhythmia 29%	8.4 (3.6) average number of drugs	Drug-related readmissions

Gustafsson et al., 2018 [27] (secondary study)	clinical pharmacists	wards at the Skellefteå County Hospital and Umeå University Hospital and to the orthopedic ward); Multicenter					dementia 20%<br>Other or unspecified dementia 47.6%	Diabetes mellitus 29%<br>Chronic obstructive pulmonary disease 8%<br>Malignant disease 13%<br>Myocardial infarction 17%<br>Stroke, past 24%		
Pfister et al., 2017 [17] (secondary study)			140 People with DRPs<br>72 People without DRPs		83.7 (6.6) (mean (SD)) People with DRPs<br>82.0 (6.3) People without DRPs	62.9% F 37.1% M People with DRPs<br>62.5% F 37.5% M People without DRPs	NR	People with DRPs:<br>Heart failure 35.7%<br>Cardiac arrhythmia 28.6%<br>Diabetes mellitus 30.7%<br>Chronic obstructive pulmonary disease 7.1%<br>Stroke, past 31.4%<br>People without DRPs:<br>Heart failure 30.6%<br>Cardiac arrhythmia 30.6%<br>Diabetes mellitus 25%<br>Chronic obstructive	People with DRPs: - 9.3 (3.4) average number of drugs at randomization<br>People without DRPs: -<br>6.8 (3.4) average number of drugs at randomization	

								pulmonary disease 8.3% Stroke, past 12%		
Abramsson et al., 2020 [28] (secondary study)			153 Patients with DRPs identified by STOPP/START 59 Patients without DRPs identified by STOPP/START		83.7 (6.3) (mean (SD)) People with DRPs identified by STOPP/START 81.6 (7.1) Patients without DRPs identified by STOPP/START	64.7% F 35.3% M People with DRPs identified by STOPP/START 57.6% F 42.4% M Patients without DRPs identified by STOPP/START	NR	People with DRPs identified by STOPP/START: - Heart failure 38.6% Cardiac arrhythmia 32.7% Diabetes mellitus 29.4% Stroke, past 26.1% Patients without DRPs identified by STOPP/START: - Heart failure 22% Cardiac arrhythmia 20.3% Diabetes mellitus 27.1% Stroke, past 16.9%	9.1 (3.5) (mean (SD)) average number of drugs prescribed- People with DRPs identified by STOPP/START 6.8 (3.2) (mean (SD)) average number of drugs prescribed- People without DRPs identified by STOPP/START	
**Germany**										
20. Wucherer et al., 2017 [43]	Retrospective study; NR; Clinical pharmacists	Community; Multicenter	446 Total (without DRP + With DRP)	Community-dwelling primary care patients screened positive for dementia	79.8 (5.4) (mean (SD))	57.6% F 42.4% M	NR	Formal diagnosis of dementia 37.2% Diagnosis of mental and behavioral disorders 25.9% Depression 16.1%	6.4 (3.2) average number of drugs prescribed	Degree of cognitive impairment

								12.1 (7.3) average comorbid diagnoses		
**Denmark**										
21. Tang et al., 2016 [47]	Prospective study; NR; Clinical pharmacists	Long-Term Care Facility (Nursing homes); Single center	12	Nursing home above 65 years of age diagnosed with dementia	87 (77-96) (mean (range))	42% M 58% F	NR	4.4 (range 2–8) average number of diagnoses per patient was	83 total number of prescription in 12 patients	Pain intensity, pain symptoms
**Hong Kong**										
22. Wong et al., 2016 [48]	Prospective study, NR; Clinical pharmacists	Hospital; Single center	54	Elderly with dementia	NR	NR	NR	NR	NR	

Note: - LTC, Long-term care facility; SD, standard deviation; F, Female; M, Male; NR, Not reported; AD, Alzheimer's disease; NMDA, N-methyl-D-aspartate; DRPs, Drug-related problems.

Table S4. Summary of interventions with reported outcomes.

	Study																					
Reported outcomes	1	2	3	4	5	6	7	8	9	10	11	12	13	14	15	16	17	18	19	20	21	22
Medication Prescription Processing (MPP)																						
1. Comprehensive Medication Management- Clinical Assessment [30,35,38,40]																						
a) Collect general medication history and other key clinical information					X	X		X	X	X	X	X	X	X	X	X	X	X	X	X	X	
b) Conduct medication review	X	X	X	X	X	X	X	X	X	X	X	X	X	X	X	X	X	X	X	X	X	X
c) Identify drug related problems (DRPs)			X		X	X					X	X			X			X	X	X		X
2. Comprehensive Medication Management- Care Plan Creation and Implementation [38,40]																						
a) Recommend interventions and solutions for DRPs			X		X					X	X	X			X	X			X		X	
3. Comprehensive Medication Management- Evaluation [30,38,40]																						
a) Follow-up and monitor results									X	X	X	X		X	X			X	X			
Educational and Advisory Services [30,38]																						
1. Secondary Patient Care Services																						
a) Perform additional patient care services, e.g. administer drugs via injection																						
2. Management of Minor Conditions																						
a) Assessment and diagnosis, triage/referral, treatment, monitor and follow-up																			X			
3. Patients, Family members, and Caregivers																						
a) Provide drug counselling services																						X
4. Health Care Professionals																						
a) Provide advice and explanations on drug information and rationale for medication use						X		X						X				X				

5. Other health care workers e.g., staff workers at facilities																						
a) Provide advice and explanations on drug information and rationale for medication use																						
Evaluation of medication use	X	X	X	X	X	X	X	X	X			X	X	X	X	X	X				X	
Cost/time effectiveness								X				X										
Drug Related Interventions																						
a) At prescriber level				X							X	X			X			X	X			
b) At patient level																						
c) At drug level			X								X	X			X			X	X			
d) Other intervention or activity										X												
Proposed intervention			49	12						3	248	543			175			261			17	
Accepted intervention			14	4						3	110	269			152			136			1	
Secondary outcomes (Cohen-Mansfield Agitation Inventory score, Neuropsychiatric Inventory score, Anticholinergic burden, Drug-related readmissions)	X						X	X	X						X			X	X	X		
Other outcomes							X	X				X					X	X			X	

Other outcomes such as quality of life, improvement, or prevention of geriatric syndromes during 1-year follow up, Pain intensity

Table S5. Overview of medication review and important clinical outcomes reported.

Study	Group	Before medication review	After medication review	Important outcomes reported
Wilchesky et al., 2018 [36]	Total number of regular medications	372	327	A significant 12.1% reduction (OR: 0.81; 95% CI: 0.70–0.92) in overall medication burden
	Total number of "sometimes" appropriate medications	194	167	Decreased (from 194 pre to 167 post-intervention)
	The mean number of regular medications per resident	7.86 (3.78) (mean (SD))	6.82 (3.75) (mean (SD))	Decreased from 7.86 to 6.81 (p = 0.007))
Dong et al., 2021 [20]	Proportions of nonadherent beneficiaries	Intervention: - Medication for Diabetes 13.1% Medication for Hypertension 16.39% Medication for Hyperlipidemia 18.69% Comparison: - Medication for Diabetes 10.84% Medication for Hypertension 13.57% Medication for Hyperlipidemia 16.06%	Intervention: - Medication for Diabetes 9.78% Medication for Hypertension 12.5% Medication for Hyperlipidemia 11.72% Comparison: - Medication for Diabetes 12.08% Medication for Hypertension 17.25% Medication for Hyperlipidemia 17.83%	Following a medication review, the percentage of non-adherent beneficiaries in the intervention group for each prescription category reduced, but they grew in the comparison group over time.
Pearson et al., 2021 [38]	180-day reduction in baseline PIM usage	1.5 PIMs per patient	0.9 PIMs per patient	Decrease from 1.5 PIMs per patient to 0.9 PIMs per patient in the patients living with dementia group
Aziz et al., 2018 [49]	Average number of prescriptions per patient	10.88 (1.27)- first audit	10.15 (0.58)- re-audit	The average number of prescriptions per patient significantly decreased, according to the results of the t-test (8% reduction), t (1) = 28.808, P = 0.022, 95% CI = 5.877–15.153.
	Number of patients	51/58- first audit	39/47- re-audit	No difference in the number of patients receiving polypharmacy, t (1) = 7.500, P =

	receiving polypharmacy			0.084. The audit revealed that polypharmacy has decreased overall by 24%.
	Average comorbidities per patient	6.23 (1.52)- first audit	5.73 (1.02)- re-audit	The average number of comorbidities between the two audits significantly decreased, according to the t-test results (7% reduction), t (1) = 23.920, P = 0.027, 95% CI = 2.803–9.157.
Ballard et al., 2016 [31,32]	Antipsychotic use by patients	20- Residents on anti-psychotic review<br>20- Residents not on anti-psychotic review	13- Residents on anti-psychotic review<br>23- Residents not on anti-psychotic review	Overall, the review group's use of antipsychotics was much lower than that of the non-review group (odds ratio 0.17, 95%CI 0.05 to 0.60, p=0.006)
	Quality-of-life score for people with dementia (proxy) (DEMQOL Proxy)	106.51 (9.14) Residents on antipsychotic review<br>102.69 (15.22) Residents not on antipsychotic review	102.11 (13.41) Residents on antipsychotic review<br>105.79 (10.53) Residents not on antipsychotic review	People receiving antipsychotic review showed a 4.54 (95% confidence interval (CI) 9.26 to 0.19) point worsening (p= 0.06) in their DEMQOL-Proxy scores, which approached statistical significance.
Massot et al., 2019 [37]	Number of psychotropic drugs prescribed	636	458	Reduced by 28% (from 636 before to 458 after the intervention).
	Mean number of psychotropic drugs prescribed per patient	2.71 (1.47)	1.95 (1.24) 1-month postintervention<br>2.06 (1.36) 6-month postintervention	Decreased from 2.71 at baseline to 1.95 at 1-month postintervention and 2.01 at 6 months (p < 0.001 for both time points). Antipsychotics were the drug class showing the highest reduction rate (49.66%)
Hernandez et al., 2020 [45]	PRISMA extension for scoping reviews by Medication Appropriateness Index (MAI) mean score	4 (4.6)	0.5 (2.6)	Significant differences (p<0.001) between the mean (SD) MAI scores at admission and post-intervention (4 (4.6) vs 0.5 (2.6))
	mean (SD) anticholinergic burden per patient	1.38 (0.7)	1.08 (0.7)	Statistically significant differences were found between pre- and post-intervention (p1 was 30 (DBI range 0.3–2.6).
	the number of patients who	44 (DBI range 0.3–3)	30 (DBI range 0.3–2.6)	

	presented with an anticholinergic burden >1 (considered high-risk burden limit)			
Molist et al., 2014 [14]	average of medications per person	7.27 prior to hospitalization	4.8 at discharge	66.85% reduction, (P < 0.05)
Gustafsson et al., 2017 [17,26-28]				multiple Cox regression model revealed that after adjustment for heart failure, the intervention significantly reduced the risk of drug-related readmissions (HR 0.49, 95% CI 0.27–0.90, p = 0.02).
	People with DRPs (n= 140) People without DRPs (n= 72)			DRPs were more common among people taking a higher number of drugs (OR, 1.255 [95% CI, 1.137- 1.385])
				DRPs were more common among people with an earlier stroke (OR, 5.042 [95% CI, 2.032-12.509]) people with heart failure (OR, 2.66 [95% CI, 1.64–4.30]), diabetes mellitus (OR, 2.32 [95% CI, 1.41–3.81]),
	Number of patients using anticholinergic drugs; NSAIDs; exposed to PIMs	15 (7.1%) at admission; 7 (3.3%) at admission; 43 (20.3%) at admission	7 (3.3%) at discharge 2 (0.9%) at discharge 30 (14.2%) at discharge	Anticholinergic drugs use decreased significantly from 7.1% to 3.3 % (p = 0.005) the use of NSAIDs decreased from 3.3% to 0.9% (p = 0.025) PIMs decreased significantly from 20.3% to 14.2% (p = 0.002)
Wucherer et al., 2017 [43]				In the multivariate Poisson regression analysis, the total number of drugs taken (b = 0.07; 95% CI: 0.05–0.09; p < 0.001) and the presence of a diagnosis of mental and behavioral disorders (b = 0.09; 95% CI: 0.03–0.15; p = 0.003) were associated with total number of DRPs (significant regression model: F(11,89) = 6.18, p < 0.001)

Note: - PIM, Potentially inappropriate medication; LWD, Living with dementia; DBI, Drug burden index; HR, Hazard ratio; OR, Odds ratio; DRPs, Drug-related problems; NSAIDs, Non-steroidal anti-inflammatory drugs
